# Evidence on collaboration of traditional and biomedical practitioners in the management of antimicrobial resistance in sub-Saharan Africa over 15 years: a systematic review protocol

**DOI:** 10.1186/s13643-021-01710-9

**Published:** 2021-05-28

**Authors:** Aganze Gloire-Aimé Mushebenge, Mukanda Gedeon Kadima, Tivani Mashamba-Thompson, Manimbulu Nlooto

**Affiliations:** 1 Discipline of Pharmaceutical Sciences, School of Health Sciences, University of KwaZulu-Natal, Durban, South Africa; 2 Discipline of Public Health Medicine, School of Nursing and Public Health, University of KwaZulu-Natal, Durban, South Africa; 3 Faculty of Health Sciences, University of Pretoria, Pretoria, South Africa; 4 Pharmacy Department, School of Healthcare Sciences, University of Limpopo, Turfloop Campus, Polokwane, South Africa

**Keywords:** Collaboration, Traditional health practitioners, Drug resistance, Traditional medicine, Absence, Conventional antimicrobial, Prevention, Effective, Sub-Saharan Africa

## Abstract

**Background:**

The overuse of prescribed antimicrobials, concurrent use of traditional medicine, and prescribed antimicrobials have led to antimicrobial resistance. The absence of collaboration between traditional health practitioners and biomedically trained healthcare professionals can contribute to antimicrobial resistance, treatment failure, overdose, toxicity, and misadministration. This scoping review explores the evidence on collaboration between traditional health practitioners and biomedically trained healthcare professionals to reduce antimicrobial resistance and treatment failure in bacterial and viral diseases.

**Methods:**

We will search for electronic databases such as Science Direct, Google Scholar, PubMed, and MEDLINE via EBSCOhost. We will also search reference lists of included studies. A two-stage mapping procedure will be carried out. Stage one (1) will consist of the title, abstracts, and full article screening, respectively. A pilot screening form guided by the defined eligibility criteria will be used. In stage two (2), data will be extracted from the included studies. Two reviewers will conduct parallel screening and data extraction. Mixed methods appraisal tool (MMAT) will be used to assess the quality of the included studies. NVIVO version 11 will be employed to aid pertinent thematic analysis. The outcomes of interest will be as follows: Primary outcome will be preventing and reducing antimicrobial resistance. The secondary effect is the effective collaboration between traditional healthcare practitioners and biomedically healthcare professionals.

**Discussion:**

This review anticipates uncovering pertinent publications reporting the evidence of collaboration between traditional health practitioners and biomedically trained healthcare professionals to reduce antimicrobial resistance in sub-Saharan Africa. The sum-up of evidence acquired from the included studies will help guide future research. The result of the study will be print and electronically exposed.

**Systematic review registration:**

PROSPERO, CRD42017072952

## Background

Traditional treatment or indigenous health system cannot be considered lower quality than the mainstream healthcare system. In contrast, it is thought to be desirable and needful in treating a range of several health troubles or difficulties that the mainstream healthcare system fails to cure sufficiently [1, 2]. In the view of Qi and Kelley [2], traditional herbal mixtures are a reliable, believable, and dignified source of health care. Bacterial and viral infections are currently common; some pathogens have become resistant to multiple antimicrobials classes [3]. Microbial adaptation allows microbes to persist despite the presence of an antibiotic or antiviral agent; this reduces the potential human health benefit derived from antibiotics and antiviral medicines [4, 5].

The ratio of traditional health practitioners and biomedically trained professionals to the general population in sub-Saharan Africa is approximately 1:500 and 1:40,000, respectively [6]. In addition to their modern biomedical treatment, up to 70% of South Africans are consulted by an estimated 200,000 indigenous traditional healers [6, 7]. Approximately 27 million South Africans, including people living with HIV/AIDS and bacteria-infected people, depend on traditional medicine (TM) for their primary health care needs [8]. STIs such as Tshofela/drop (gonorrhea), Thosola (syphilis), some other specific bacterial infections, and assumed HIV/AIDS is the most commonly treated conditions and problems by traditional health practitioners (THPs) [9]. This is probably due to the excellent accessibility of plants, affordability, the confidentiality of health information between the patient and practitioner, and the high cost of synthetic medicines [10]. Furthermore, consumers believe that certain infections such as acne, warts, shingles, and STIs cannot be treated with western medicine by biomedically trained healthcare professionals(BHPs) but rather by THPs [11, 12]. Medicinal plants are effective, cheap, readily available, and used for cultural reasons.

In South Africa, antimicrobial resistance (AMR) is hugely prevalent. Some bacteria and viruses are becoming so resistant that there is either only antimicrobial of last resort or infections are untreatable [13]. AMR in South Africa is driven by many factors such as the careless use of prescribed antimicrobials, the use of TM currently not regulated, the lack of collaboration between THPs, and BHPs, with the result of treatment failure, misadministration, interactions, and toxicity [14]. To conceptualize the conditions for collaboration between the two systems, Pretorius [15] proposed the Biomedical/Traditional Medical Relationship’s analogical model. This model shows clearly that each aspect of the traditional medicine system may be related to the western medicine system, such as mutual referral. Besides, the WHO have adopted the strategies to ensure the integration of collaboration between research institutions and THPs based on research and management of patients, and between THPs and BHPs in human immunodeficiency virus and acquired immune deficiency syndrome (HIV/AIDS) prevention and sexually transmitted infections (STIs)/tuberculosis programs [16].

Although there is an increase in TM’s use, it is not currently regulated, with the result of enhancement of the activities of standard medicines when used concurrently with TM [17]. Nascimento et al. reported that the danger of misadministration, drug interactions, overdose, and toxicity, especially the problem of drug resistance and treatment failure, can occur when TM and prescribed antimicrobials are simultaneously and indiscriminately used [18]. This review aims to map the evidence on the collaboration between THPs and BHPs to reduce antimicrobial resistance in sub-Saharan Africa.

## Method/design

### Scoping review framework

The authors will conduct a scoping review of peer-reviewed literature on the following specific points: concurrent use of traditional medicines and prescribed antimicrobials, the collaboration between THPs and BHPs, and treatment of bacterial and viral diseases. The scoping review method’s selection was to make easy the mapping of the topic under study and build evidence around the related to the subject [19]. This review will use the framework developed by Arksey and O’Malley [20]. This framework stipulated the following steps (a) identification of research questions, (b) identification of relevant studies, (c) charting the data, and (d) collation, summary, and report of findings.

### Identifying the research questions

The general research question of this study is, “What is the evidence of collaboration between THPs and BHPs in the reduction of antimicrobial resistance among people living with infectious diseases in sub-Sahara African countries?”

The specific research questions to answer the general question are as follows:

1. What is the evidence of TM’s concurrent use, prescribed ATB, and ARVs medicine for viral and bacterial infections?
2. What is the evidence of the perceptions of  THPs and BHPs about the interaction between TM and prescribed ATB and ARV medicine for viral and bacterial infections?
3. Is there a bi-directional referral of patients between THPs and BHPs to manage bacterial and viral diseases?

### Eligibility of research questions

The study will use an amended Population, Intervention, Comparison, Outcomes, and Study setting (PICOS) framework to evaluate research questions' eligibility (Table 1).

**Table 1:** PICOS framework for determination of eligibility of review question

Criteria	Determinants
Population	THPs and BHPs
Intervention	Collaboration between traditional and biomedically healthcare workers
Comparison	Absence of collaboration between THPs and BHPs
Outcomes	Primary outcome: Prevention and reduction of antimicrobial resistance Secondary outcome: Effective collaboration
Setting	Sub-Sahara Africa.

### Identification of relevant studies

Studies that utilize mixed methods, qualitative and quantitative, published in peer-reviewed journals and university research spaces, thesis and dissertations, conference papers, and government desks will be assessed as part of the grey literature. Collected data will be from January 2005 to May  2021, addressing the above research questions. Different types of study designs will be used during the process of screening data. Authors will conduct an electronic search from the following databases: Science Direct, Scopus, Web of Science and Embase, Google Scholar, PubMed, Medrxiv and MEDLINE via EBSCOhost. Authors will explore internet sites such as the World Health Organization (WHO) and government internet sites for reports and policies on the collaboration of healthcare workers, measures on antimicrobial resistance, safe use of traditional medicine, and concurrent use of traditional medicine and prescribed medicines. Through “Cited by,” other articles will also be searched in the reference lists of selected papers. The search keywords will include Collaboration, Traditional health practitioners, Drug-resistance, Traditional medicine, Absence, Conventional antimicrobial, Prevention, Effective, and sub-Saharan Africa.

### Study selection

To be sure that the included studies have the specific information according to the eligibility criteria, they should respond to the evidence of collaboration between THPs and BHPs in reducing antimicrobial resistance and treatment failure in bacterial and viral diseases.

#### Inclusion criteria

For the inclusion of publications in this study, they should match with the undermentioned criteria:

1. There will be no language restriction in the inclusion of studies. The authors will request English or French versions of Italian, German, Chinese, and Portuguese exciting materials.
2. Focus on strategies of collaboration between THPs and BHPs
3. Publications from January 2005 to May 2021
4. Report on cases of treatment failure, drug interactions, drug resistance, and antimicrobial stewardship
5. Publications on the use of traditional medicine in conjunction with prescribed antimicrobials by community members of 18 years and above
6. Reports on death cause worldwide, with particular emphasis on sub-Saharan Africa

#### Exclusion criteria

Studies will be excluded if they meet the following characteristics:

- Articles published before 2005 and after May 2021
- Articles that do not report on TM's use for the management of bacterial and viral diseases
- Articles that report on other diseases than infectious diseases

### Search strategy

A pilot study will be carried out to check the chosen studies’ appropriateness, keywords, and databases. Selected articles will be shared between two reviewers using research manager software such as Endnote library. According to the eligibility criteria, both the first and second reviewers will conduct a comprehensive title screening. Eligible publications will be exported using Endnote management software. Articles duplication will be checked using the EndNote program. Eligible studies will be searched from January 2005 to May 2021 through Google Scholar, Medline (PubMed), Medrxiv, and the Cochrane Library, published in English or French. This review will refer for its search strategy to the following terms: (Collaboration OR cooperation OR coaction) AND (Traditional healer practitioners OR Traditional healthcare practitioners OR Traditional healers OR Traditional practitioners) AND (Biomedically Professional doctors OR Healthcare workers OR Healthcare professionals OR) AND (Concurrent use OR concomitant use OR simultaneous use OR use in association) AND (Mainstream healthcare system OR orthodox therapy OR modern therapy OR western medicine OR Conventional therapy) AND (Traditional herbal mixtures OR herbal medicines OR herbal concoctions OR herbal teas OR herbal formulations OR Medicinal plant OR Traditional medicine) AND (Drug-resistance OR Microbial adaptation OR antimicrobial resistance) AND (Infectious diseases OR viral infectious diseases OR contagious bacterial diseases) AND (Mutual referral OR bi-directional).

Full articles and abstracts of studies will be screened according to the eligible criteria. The authors will consider a third reviewer in case of non-accordance between the two previous reviewers. In case of difficulty to find some articles, the authors will need the assistance of the UKZN library. However, for authors whose publications will be cited and challenging to retrieve, they will be asked for assistance through a correspondence letter. If they do not respond to the correspondence, then their articles will be excluded. After the selection process of publications, an appropriate checklist to critically appraise each study design will be applied and conducted in pairs. Table 2 presents the electronic search engine for studies found using the keywords.

**Table 2:** Electronic search record

Date	Keywords	Search engine	Number of studies found
19 April 2021	Collaboration, Traditional Health Practitioners, Drug-resistance, traditional medicine, Absence, Conventional antimicrobial, Prevention, Effective, sub-Saharan Africa.	Google Scholar	1160
23 April 2021	(Traditional herbal mixtures OR herbal medicines OR herbal concoctions OR herbal teas OR herbal formulations OR Medicinal plant OR Traditional medicine)	PubMed	1448

### Charting the data

Table 3 presents the flow or the charting of included studies. A data charting form will be conceived and piloted. Variables to have to summarize the included articles are shown in Table 3.

**Table 3:** Form for data charting

Author and date Article or study title Journal full reference Aims or main research question Characteristics of participants Recruitment context (e.g., where participants were recruited). Sampling method Study method or design Theoretical background Data collection (what data collection methods were used?) Data analysis (how was the data analyzed?) Intervention Intervention outcome Most relevant findings Conclusions Comments	

### Collating, summarizing, and reporting the findings

This study aims to map the evidence of collaboration between THPs and BHPs in reducing antimicrobial resistance and treatment failure in bacterial and viral diseases in sub-Saharan Africa and summarizing the results as found from the included studies. Following data extraction, thematic content analysis will be carried out to code the data according to the following themes: types of interactions registered in publications; causes of treatment failure, approach system, and medicinal plants used by THPs to treat infectious diseases; barriers and facilitators towards collaboration between THPs and BHPs in the management of infectious diseases which may lead to antimicrobial resistance; and types of infectious disease not cured by Western medicine. The emerging theme will also be coded. NVIVO software version 11 will be employed to assist with the coding of the themes [21]. The process will be done as follows:

- Coding data from the included articles
- Categorizing the codes into major themes
- Displaying the data
- Identification of critical patterns in the data and identification of subthemes
- Summarizing

### Quality appraisal

The mixed-method appraisal tool (MMAT)-Version 2011 will evaluate the quality of the included studies [22]. This tool will assess the appropriateness of the study’s aim, adequacy and methodology, study design, participant recruitment, data collection, data analysis, presentation of findings, and authors’ discussions and conclusions. Selected studies will be scored based on a criterion that will use a score to describe them (50% and above). The strength of the body of evidence in the systematic review will be assessed using GRADE.

### Synthesis

The details have been provided in this synthesis sub-section and read as follows: Data will be analyzed using a narrative approach, specifically thematic synthesis. The resulting themes will be analyzed and critically examined in relationship with the research questions. Since the primary goal of this systematic review is to review and synthesize published studies focusing on evidence of collaboration between THPs and BHPs in the reduction of antimicrobial resistance among people with infectious diseases in sub-Sahara African countries, all data derived from the selected articles will be presented in a text and summary tables in a narrative format. The publications to be included in this study are intended to be diverse. They will be carefully examined, and the limitations of each will be identified (i.e., quality assessment). Furthermore, the entire data extraction and synthesis process will be meticulously recorded. Meta-analysis can only be conducted in case the included studies are sufficiently homogeneous when it comes to their design, the population, interventions, and comparators, reporting the same outcome measures (PICO). Reviewers will explore the meanings of the results in reference to the aim of the research and the implications of these results for the forthcoming research, practice, and policy. The present protocol will use the developed guidelines for reporting known as the Preferred Reporting Items for Systematic Reviews and Protocol Meta-Analyses 2015 (PRISMA-P 2015). The PRISMA-P consists of a checklist of 17 elements designed to promote the planning and reporting of a robust systematic review protocol [23].

## Discussion

This scoping review will be carried out as the first part of a more extensive study on the evidence of collaboration between THPs and BHPs in reducing antimicrobial resistance and treatment failure in bacterial and viral diseases in sub-Saharan Africa. This review will identify types of interactions registered in publications, causes of treatment failure, approach system, medicinal plants used by THPs to treat infectious diseases, barriers and facilitators towards collaboration between THPs and BHPs, and types of infectious diseases cured by western medicine. Although there is a growing acknowledgment that healthcare systems are encouraging collaboration between THPs and BHPs [24–27], there is a lack of knowledge about the partnership between THPs and BHPs about antimicrobial resistance, treatment failure, or other interactions. Also, there are challenges related to the availability of relevant full-text articles.

Articles that report on other diseases than infectious diseases will be excluded because this study is focused on the use of prescribed antimicrobials and TM in the management of infectious diseases. This review excludes all tasks that do not report TM’s help to manage bacterial and viral infections. All the reports on deaths that are not caused by infectious diseases and those that are not reporting cases of interaction either in TM alone, prescribed antimicrobials alone, or in the concurrent use of both TM and prescribed antimicrobials will be excluded.

Results from this study will be of benefit to researchers by highlighting gaps in evidence that may need further investigation. Study findings will be disseminated in peer-reviewed publications.

## Data Availability

All data generated or analyzed during this study will be included in the published scoping review.

## Abbreviations

THPs: Traditional healer practitioners
BHPs: Biomedically healthcare professionals
PICOS: Population, intervention, comparison, outcomes, and study setting
MMAT: Mixed Methods Appraisal Tool
HIV/AIDS: Human immunodeficiency virus/acquired immunodeficiency syndrome
TM: Traditional medicine

## References

[1] Organization WH (2004). WHO guidelines on developing consumer information on proper use of traditional, complementary and alternative medicine.

[2] Qi Z, Kelley E (2014). The WHO traditional medicine strategy 2014–2023: a perspective. Science.

[3] Ledingham JG, Warrell DA (2000). Concise Oxford textbook of medicine: Oxford university press.

[4] Hunt G, Ledwaba J, Salimo A, Kalimashe M, Singh B, Puren A, Morris L (2013). Surveillance of transmitted HIV-1 drug resistance in 5 provinces in South Africa in 2011. Communicable Diseases Surveillance Bull.

[5] Lathers CM (2002). Clinical pharmacology of antimicrobial use in humans and animals. The J Clin Pharmacology.

[6] Richter M (2004). Traditional healing and human rights in South Africa. In: XV International AIDS Conference, Bangkok.

[7] Van Wyk B-E, Gericke N (2000). People's plants: A guide to useful plants of Southern Africa: Briza Publications.

[8] Street R, Stirk W, Van Staden J (2008). South African traditional medicinal plant trade—challenges in regulating quality, safety and efficacy. J Ethnopharmacol.

[9] Peltzer K (2001). An investigation into the practices of traditional and faith healers in an urban setting in South Africa. Health SA Gesondheid.

[10] Ballabh B, Chaurasia O, Ahmed Z, Singh SB (2008). Traditional medicinal plants of cold desert Ladakh—used against kidney and urinary disorders. J Ethnopharmacol.

[11] de Wet H, Nkwanyana MN, van Vuuren SF (2010). Medicinal plants used for the treatment of diarrhoea in northern Maputaland, KwaZulu-Natal Province, South Africa. J Ethnopharmacology.

[12] York T, De Wet H, Van Vuuren S (2011). Plants used for treating respiratory infections in rural Maputaland, KwaZulu-Natal, South Africa. J Ethnopharmacol.

[13] Sadarangani SP, Estes LL, Steckelberg JM. Non–anti-infective effects of antimicrobials and their clinical applications: a review. In: Mayo Clinic Proceedings: 2015: Elsevier; 2015;90(1):109–27. 10.1016/j.mayocp.2014.09.006. 10.1016/j.mayocp.2014.09.006 25440726

[14] Fennell C, Lindsey K, McGaw L, Sparg S, Stafford G, Elgorashi E, Grace O, Van Staden J (2004). Assessing African medicinal plants for efficacy and safety: pharmacological screening and toxicology. J Ethnopharmacol.

[15] Pretorius E (1991). Traditional and modern medicine working in tandem. Curationis.

[16] Busia K, Kasilo OM. Collaboration between traditional health practitioners and conventional health practitioners: some country experiences. Afr Health Monit. 2010;13:40–6.

[17] Kamatou G, Viljoen A, Van Vuuren S, Van Zyl R (2006). In vitro evidence of antimicrobial synergy between *Salvia chamelaeagnea* and *Leonotis leonurus*. South Afr J Botany.

[18] Nascimento GG, Locatelli J, Freitas PC, Silva GL (2000). Antibacterial activity of plant extracts and phytochemicals on antibiotic-resistant bacteria. Braz J Microbiol.

[19] Colquhoun HL, Levac D, O'Brien KK, Straus S, Tricco AC, Perrier L, Kastner M, Moher D (2014). Scoping reviews: time for clarity in definition, methods, and reporting. J Clin Epidemiol.

[20] Arksey H, O'Malley L (2005). Scoping studies: towards a methodological framework. International journal of social research methodology.

[21] Castleberry A (2014). NVivo 10 [software program]. Version 10. QSR International: 2012. AJPE.

[22] Pluye P, Robert E, Cargo M, Bartlett G, O'cathain A, Griffiths F, Boardman F, Gagnon M-P, Rousseau M (2011). Proposal: A mixed methods appraisal tool for systematic mixed studies reviews.

[23] Moher D, Shamseer L, Clarke M, Ghersi D, Liberati A, Petticrew M, Shekelle P, Stewart LA: Preferred reporting items for systematic review and meta-analysis protocols (PRISMA-P) 2015 statement. Syst Rev. 2015;4(1):1–9. 10.1186/2046-4053-4-1. 10.1186/2046-4053-4-1 PMC4320440 25554246

[24] Bedwell WL, Wildman JL, DiazGranados D, Salazar M, Kramer WS, Salas E (2012). Collaboration at work: An integrative multilevel conceptualization. Human Resource Management Review.

[25] Kozlowski SW, Chao GT, Grand JA, Braun MT, Kuljanin G (2013). Advancing multilevel research design: Capturing the dynamics of emergence. Organ Res Methods.

[26] Nancarrow SA, Booth A, Ariss S, Smith T, Enderby P, Roots A (2013). Ten principles of good interdisciplinary team work. Human Resources Health.

[27] Walsh JA, Warren KS (1980). Selective primary health care: an interim strategy for disease control in developing countries. Soc Sci Med Part C Med Econ.

